# Multidrug-Resistant Bacterial Pathogens and Public Health: The Antimicrobial Effect of Cyanobacterial-Biosynthesized Silver Nanoparticles

**DOI:** 10.3390/antibiotics11081003

**Published:** 2022-07-26

**Authors:** Nermin A. El Semary, Esam M. Bakir

**Affiliations:** 1 Al Bild Bank Scholarly Chair for Food Security in Saudi Arabia, Deanship of Scientific Research, Vice Presidency for Graduate Studies and Scientific Research, King Faisal University, Al-Ahsa 31982, Saudi Arabia; 2 Biological Sciences Department, College of Science, King Faisal University, Al-Ahsa 31982, Saudi Arabia; 3 Botany and Microbiology Department, Faculty of Science, Helwan University, Ain Helwan, Cairo 11795, Egypt; 4 Chemistry Department, Faculty of Science, Ain Shams University, Al-Abassia, Cairo 11566, Egypt; ebakir@kfu.edu.sa; 5 Chemistry Department, College of Science, King Faisal University, Al-Ahsa 31982, Saudi Arabia

**Keywords:** cyanobacteria, DLS, FTIR, molecular characterization, silver nanoparticles, size manipulation

## Abstract

Background: Cyanobacteria are considered as green nano-factories. Manipulation of the size of biogenic silver nanoparticles is needed to produce particles that suit the different applications such as the use as antibacterial agents. The present study attempts to manipulate the size of biosynthesized silver nanoparticles produced by cyanobacteria and to test the different-sized nanoparticles against pathogenic clinical bacteria. Methods: *Cyanothece*-like. coccoid unicellular cyanobacterium was tested for its ability to biosynthesize nanosilver particles of different sizes. A stock solution of silver nitrate was prepared from which three different concentrations were added to cyanobacterial culture. UV-visible spectroscopy and FTIR were conducted to characterize the silver nanoparticles produced in the cell free filtrate. Dynamic Light Scattering (DLS) was performed to determine the size of the nanoparticles produced at each concentration. The antimicrobial bioassays were conducted on broad host methicillin-resistant *Staphylococcus aureus* (MRSA), and *Streptococcus* sp., was conducted to detect the nanoparticle size that was most efficient as an antimicrobial agent. Results. The UV-Visible spectra showed excellent congruence of the plasmon peak characteristic of nanosilver at 450 nm for all three different concentrations, varying peak heights were recorded according to the concentration used. The FTIR of the three solutions revealed the absence of characteristic functional groups in the solution. All three concentrations showed spectra at 1636 and 2050–2290 nm indicating uniformity of composition. Moreover, DLS analysis revealed that the silver nanoparticles produced with lowest concentration of precursor AgNO_3_ had smallest size followed by those resulting from the higher precursor concentration. The nanoparticles resulting from highest concentration of precursor AgNO_3_ were the biggest in size and tending to agglomerate when their size was above 100 nm. The three types of differently-sized silver nanoparticles were used against two bacterial pathogenic strains with broad host range; MRSA-(Methicillin-resistant *Staphylococcus aureus*) and *Streptococcus* sp. The three types of nanoparticles showed antimicrobial effects with the smallest nanoparticles being the most efficient in inhibiting bacterial growth. Discussion: Nanosilver particles biosynthesized by *Cyanothece*-like cyanobacterium can serve as antibacterial agent against pathogens including multi-drug resistant strains. The most appropriate nanoparticle size for efficient antimicrobial activity had to be identified. Hence, size-manipulation experiment was conducted to find the most effective size of nanosilver particles. This size manipulation was achieved by controlling the amount of starting precursor. Excessive precursor material resulted in the agglomeration of the silver nanoparticles to a size greater than 100 nm. Thereby decreasing their ability to penetrate into the inner vicinity of microbial cells and consequently decreasing their antibacterial potency. Conclusion: Antibacterial nanosilver particles can be biosynthesized and their size manipulated by green synthesis. The use of biogenic nanosilver particles as small as possible is recommended to obtain effective antibacterial agents.

## 1. Introduction

The green synthesis of nanoparticles is ideal as it does not cause pollution and is of minimal cost. In addition, allows nanoparticles production in large quantities with no toxic by-products [1]. Inorganic nanoparticles of noble metals such as gold and silver nanoparticles are increasingly used in biology and medicine due to their distinctive characteristics such as ease of use, good functionality, biocompatibility and ability to target specific cells [2]. In regard to their production, cyanobacteria are considered an active source of nanomaterials [3,4]. Several cyanobacterial genera are reported to produce nanoparticles including; *Anabaena*, *Calothrix*, and *Leptolyngbya* which actively produced Au, Ag, Pd, and Pt nanoparticles. These particles are naturally released in the culture medium and stabilized by algal polysaccharides/peptides that enable easy recovery. The size of the recovered particles and yield depend on the cyanobacterial genus [5]. The mechanisms by which those nanoparticles are produced were recently reviewed [6] and their biosynthesis was classified into extracellular and intracellular. In intracellular biosynthesis, ions are reduced by electrons in the electron transport systems that are involved in photosynthesis and respiration. Enzymes such as NADH-dependent reductases are mostly involved in electron transport and redox reactions in the cytoplasm, thylakoid membranes, and plasma membrane [7,8]. Extracellular synthesis involves cellular exudates such as pigments, proteins, enzymes, hormones, and ions which play an important role in the reduction and capping process of nanoparticles [9,10]. Biomolecules such as NADH-reductases and sulfur-containing proteins in the cell-free supernatant are important in bio-reduction of nanoparticles [11,12,13]. Due to the antibacterial action of silver nanoparticles, they are incorporated in footwear, cosmetics, wound dressings and plastics [11]. The antimicrobial effect of silver nanoparticles, biosynthesized by algae against human bacterial pathogens has been previously reported [14,15]. The cyanobacterium under study is unicellular, photosynthetic prokaryotic microorganism. It can be grown easily with minimal growth requirements in the presence of light source. This ease of growth ensures a continuous supply of cells capable of biosynthesizing nanosilver particles. In addition, this cyanobacterium is Gram-negative which means it has a large outer lipid membrane made of fatty acids that are important functional molcules in the binding and possibly reduction of nanosilver ions from a solution [16]. The *Cyanothece* genus is a quite unique as it is able to undergo diurnal cycle of photosynthesis in the day and perform nitrogen fixation in the night. Both of the two are reducing processes which only indicates its strong reducing abilities [17]. Indeed, *Cyanothece* spp. showed unique reducing ability and ease of manipulation for the production of nanogold particles of different sizes [4]. Here we isolated and used *Cyanothece*-like cyanobacterial strain in an attempt to biosynthesize silver nanoparticles to be used as antimicrobial agents. Analysis by Ultra violet-visible (UV-vis.) spectroscopy and Fourier Transmission Infrared spectroscopy (FTIR) were carried out for all different Silver nanoparticles (AgNPs) samples. In the UV-vis. spectroscopy, surface plasmon resonance (SPR) was recorded and indicated the specific vibration modes of electrons limited by the size and shape of the nanoparticles. [18]. Fourier transmission Infrared spectroscopy will also be performed to detect any functional groups associated with silver nanoparticles [14] in the water-based cell-free filtrate. Moreover, the Dynamic Light Scattering will be used for accurate determination of the size of the three AgNPs samples [19]. The AgNPs samples were tested in antimicrobial bioassays against bacterial pathogens. It is noteworthy that the extent of antimicrobial activity of biosynthesized nanosilver particles against bacterial pathogens appeared to be linked to particles’ size [20]. Unfortunately, the size manipulation of silver nanoparticles biosynthesized using cyanobacteria has been scarcely studied. On the other hand, size-manipulation of gold nanoparticles that were biologically synthesized was successfully achieved [4]. Here, we provide a simple protocol for the manipulation of the size of silver nanoparticles produced and we investigated the antibacterial impact of differently-sized nanosilver particles on two multi-drug resistant clinical pathogenic bacteria. 

## 2. Methods and Materials

### 2.1. Cyanobacterial Culture Establishment and Identification

The *Cyanothece*-like coccoid unicellular cyanobacterium used in the experiment was originally isolated from a rice-field in Al Ahsa, Eastern Province, KSA. Water samples were taken from canals filled with irrigation water. The samples were centrifuged at 3000 rpm for 10 min to eliminate contaminating bacteria by discarding the supernatant under a 12:12 h (light:dark) cycle at ambient temperature. The biomass pellet was streaked on agar plates based on BG11 growth medium. Cultures were kept under 12:12 h (light:dark) cycle at ambient temperature. The green colonies were picked and re-streaked on agar plates and then examined by light microscopy for morphological description. Liquid cultures of BG11 were inoculated with pure colonies and left to grow as monoalgal cultures. The cultures were subjected to antibiotic treatment using Ampicillin (200 µL/L) and left in the dark for two days to kill heterotrophic bacteria and then brought back to light and centrifuged. The supernatant was discarded and the biomass was re-suspended in sterile water for washing. Centrifugation and washing steps were repeated then the biomass was inoculated into fresh sterile BG11 medium, left to grow and checked microscopically to ascertain that axenic cyanobacterial culture was established for use in experiments.

### 2.2. Preparation of Nanosilver Particles

A stock solution of 10 mM silver nitrate (Sigma, Aldrich) was prepared. Three different volumes of the same stock were prepared (2 mL, 1 mL and 0.2 mL) and added to a cyanobacterial biomass (0.5 g fresh weight in 5 mL BG 11 growth medium) then, completed with distilled water to obtain a 20 mL total volume. The solutions were left for three days, the colour change was observed from the onset of the experiment. The external solution containing nanosilver was purified from the cells through filtration using Millipore filters of diameter size of 0.2 µm. This tiny pore size excluded all aggregates, cells, and bulky cellular components. The cell-free filtrate was watery in nature, as the stock precursor AgNO_3_ was prepared in distilled water and the cyanobacterium was cultured in a water-based mineral growth medium (BG11). Siver nanoparticles were synthesized in a mixture containing distilled water, +5 mL of the cyanobacterial culture, and +10 mM AgNO_3_ (added in three different volumes; 0.2, 2, and 1 mL). We assessed the nanoparticles in the cell-free supernatant after filtration through Millipore filters of pore diameter size of 0.2 µm.

### 2.3. UV–Visible Spectroscopic (UV–Vis) Analysis

The initial characterization of the silver nanoparticles was performed using a UV–vis spectrophotometer (Genesys10S UV–visible double beam spectrophotometer). The scans were recorded at room temperature using 1 mL of each nanosilver concentration in the range of 200 nm to 700 nm.

### 2.4. FTIR

Infrared spectrometric analysis of silver nanoparticles was performed on cell-free supernatant containing nanosilver using Fourier-Transform infrared spectrometer (FT-IR, Agilent Cory 630, Agilent Technologies, Santa Clara, CA, USA). A control sample with no silver nitrate was also used.

### 2.5. Dynamic Light Scattering Analysis

The size of the silver nanoparticles was studied by Dynamic light scattering (DLS), using Dual Scattering Particle Size Analyzer (cilas, Nano DS). Each sample was analyzed in triplicates at 25 °C with scattering angle 60°. Deionized water was used as the dispersal medium. The integration time was 30 min and the algorithm used was cumulative. The samples were loaded into quartz microcuvettes, and replicate measurements were recorded, of which the mean was calculated.

### 2.6. Antibacterial Bioassay

The antibacterial activity of the three solutions of the nanosilver particles was assessed against freshly sub-cultured bacteria originally isolated from clinical samples at the College of Medicine, King Faisal University. The two bacterial isolates were identified as Gram-positive MRSA-Methicillin-resistant *Staphylococcus aureus* and a *Streptococcus* sp. and supplied by Dr Munirah Aldayel, King Faisal University. The sensitivity of pathogenic strains to nanosilver was assessed by modified Kirby-Bauer Disk Diffusion Susceptibility method [21]. Sterile paper discs (6 mm in diameter) were saturated with 30 µL of nanosilver at the three concentrations examined. The discs were dried and placed on the surface of nutrient agar medium inoculated with a bacterial-suspension and kept for 24 h in an incubator at 37 °C. A positive control disc containing 30 µL antibiotic Chloramphenicol was also used. The diameter of the inhibition zones (mm) was measured in triplicates and the average and standard deviation were recorded [22]. As a negative control the supernatant without AgNO_3_ was also used. The MIC/MBC was also determined to verify the bacteriostatic/bactericidal effect of the three types of the differently-sized nanoparticles. 

## 3. Results

### 3.1. Description of Cyanobacterial Strain

The unicellular cyanobacterium tested appeared in pairs after division. No colonies form as there is no common mucilaginous envelope. Cells are coccoid and usually bright blue-green. All of these characteristics are typical of the genus *Cyanothece*. Molecular characterization revealed only 88% of similarity to *Cyanothece* sp. Due to the lack of sufficient diagnostic phenotypic characters of the coccoid cyanobacterium, it was designated *Cyanothece*-like cyanobacterium. 

### 3.2. Preparations of Different Silver Nanoparticles Samples

The culture inoculum contained 4 × 10^4^ cells/mL taken from one month old culture, The three different concentrations of silver nitrate applied to the cyanobacterial culture were; 1 × 10^−4^ M, 5 × 10^−4^ M and 1 × 10^−3^ M. The samples showed a gradual color change from faint brown to, light brown and then to dark brown, corresponding to the volumes of the precursor materials, i.e., 0.2, 1, and 2 mL, respectively. Each mixture was left in at room temperature, and the cell-free supernatant was taken from external solution of the cultures after incubation period of three days. The solution was microfiltered using Millipore filters of pore diameter size of 0.2 µm. The cell-free filtrates of the three samples were used in further analyses.

### 3.3. UV-Visible Spectroscopy

**Figure 1:**
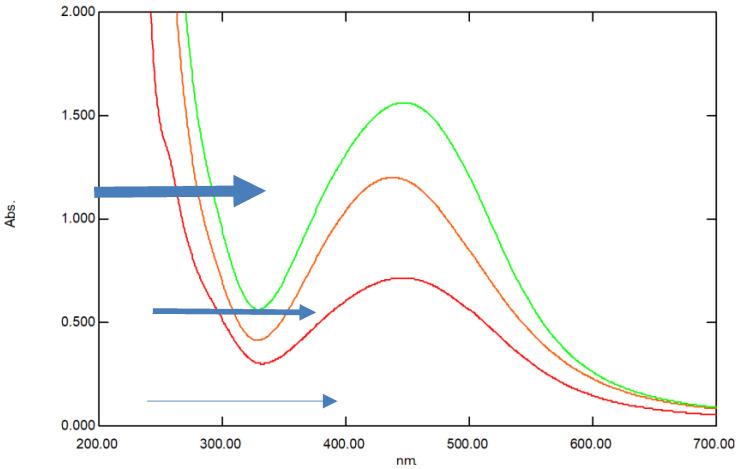
UV-Visible spectrum of the three samples of AgNPs with different heights and width according to the nanoparticle size. The lowest peak (red) denoted by the lower thin arrow belongs to the smallest-sized AgNP, the middle peak (red) denoted by the middle arrow belongs to the medium-sized particles and the highest peak (green) belongs to the largest sized silver nanoparticle. The X-axis denotes the wavelength whereas y-axis denotes the absorbance.

Nano-silver particles were successfully synthesized both intra and extra-cellularly. The extracellular formation of AgNPs was confirmed by UV-Vis absorbance spectra of AgNPs of the three samples with the highest peak belonging to highest concentration of nanosilver followed by the medium concentration and lowest concentration, respectively (Figure 1). All the three concentrations of biogenic nanosilver showed a strong specific peak for the synthesized AgNPs at 450 nm (Figure 1). There was congruence for the peaks detected for the three concentrations. This coincided with the plasmon resonance characteristic of nanosilver particles [23]. 

### 3.4. FTIR Spectroscopy

**Figure 2:**
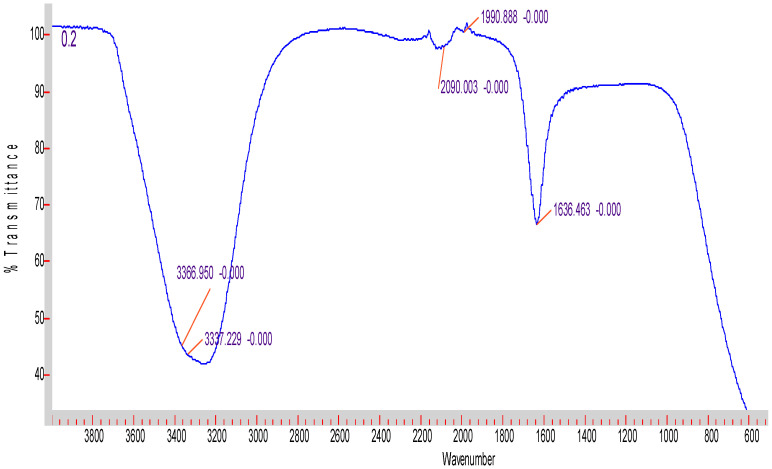
FTIR of the smallest−sized AgNPs. The x-axis denotes the wavenumber, The y-axis denotes % transmittance.

The FTIR spectra of the three samples of AgNPs had the same pattern exemplified in Figure 2 and clearly exhibited the characteristic signals of AgNPs at 3356–3350 cm^−1^ overlapping with OH signal. At 1636–1637 cm^−1^ there was a clear signal corresponding to C-H stretching [24]. No other functional groups were detected in all samples. (Figure 2 and Supplementary Materials Figures S1 and S2).

### 3.5. Dynamic Light Scattering

**Figure 3:**
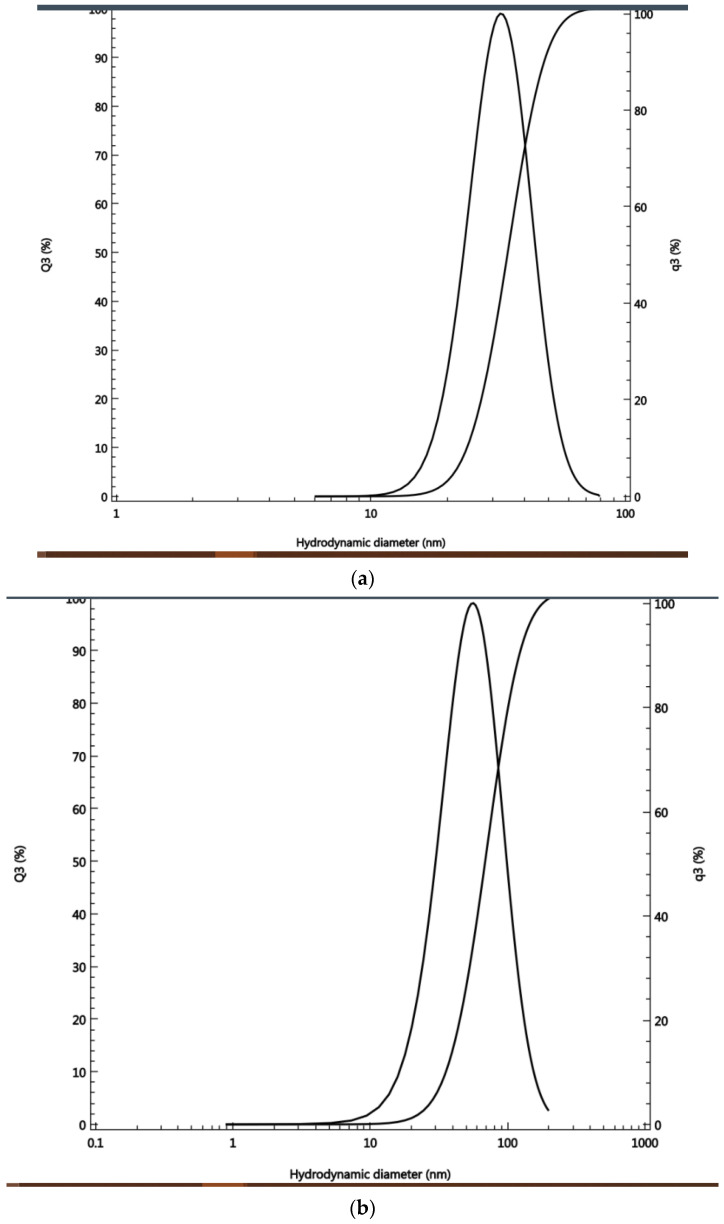

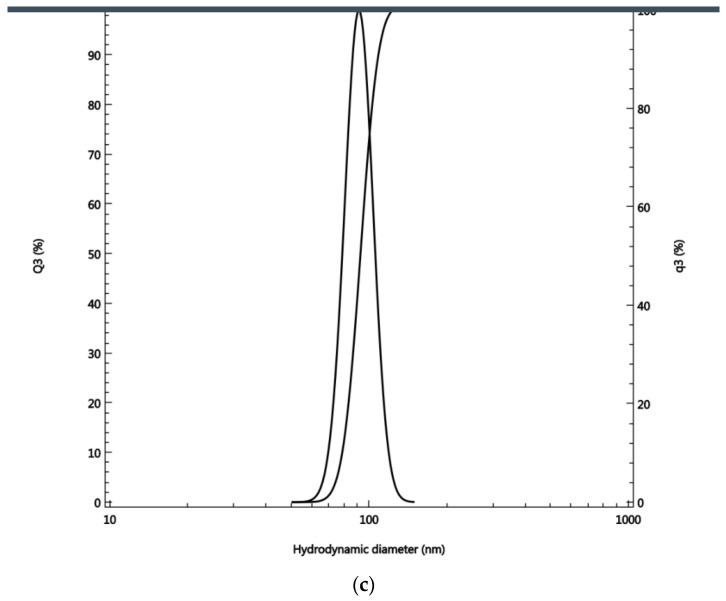
Hydrodynamic diameter of the (**a**) smallest-, (**b**) medium-, (**c**) largest-sized AgNPs.

According to the DLS analysis, the size range for the smallest-sized silver nano-particles (1 × 10^−4^ M) was 23–47 nm, with an average of 33.9 nm. The coefficient of var-iation (%) was 27.5, the polydispersity index (%) was 71.7, and rms was 0.36329. The size of the medium-sized silver nanoparticles (5 × 10^−4^ M) was in the range of 35–122 nm, with an average of 67 nm. The coefficient of variation (%) was 47, the polydisper-sity index (%) was 130.5, and rms was 0.02417. The size range for the highest-sized sil-ver nanoparticles was 78–108 nm, with some nanoparticles above 100 nm (Figure 3), with an average of 92.5 nm. The coefficient of variation (%) was 12.5, the polydispersi-ty index (%) was 32.3 (Figure 3)

### 3.6. Antimicrobial Bioassay

The three different nanosilver concentrations showed strong antimicrobial effects, with the smallest-sized nanoparticles being the most efficient in inhibiting bacterial growth, showing an inhibition zone diameter of 1.8 cm for the *Streptococcus* sp. and of 1.6 cm for *Staphylococcus aureus* (MRSA). The second potent antimicrobial concentration was that of medium-sized nanosilver particles (derived from 1 mL of AgNO_3_ solution), with an inhibition zone of 1.4 cm for *Staphylococcus aureus* (MRSA) and of 1.3 cm for the Streptococcus sp. The least effective were the largest sized-nanosilver particles (derived from 2 mL of AgNO_3_ solution), with an inhibition zone of 1.2 cm for the *Streptococcus* sp. and of 1.1 for *Staphylococcus aureus* (MRSA). (Table 1) The bactericidal and bacteriostatic effects for the three sized AgNPs were verified using an antimicrobial bioassay and calculating the MIC/MBC in µg/mL (Table 2).

### 3.7. Antimicrobial Bioassay Determning MIC/MBC in µg/mL

The minimum inhibitory concentration (MIC) and minimum bactericidal concentration (MBC) for the silver nanoparticles of different particle size were investigated. The nanoparticles exhibited both bacteriostatic and bactericidal capabilities (Table 2). The lowest MIC and MBC values were for the smallest-sized silver nanoparticles, indicating their effectiveness at the lowest concentration.

## 4. Discussion

The ability of cyanobacteria to bind bulk ions from solutions followed by further reduction and nano-formation is mainly related to surface entities on cyanobacteria as well as to the polysaccharide sheath present on the cyanobacterial surface and, sometimes, in solution [3]. Indeed, our previous work showed the biosynthetic ability of cyanobacteria for gold nanoparticles [3], with the possibility of customized biosynthesis as well [4]. Cell-free media were found to be required for the synthesis of nanoparticles, as their content of enzymes, antioxidants, and phenolic and ions facilitates the bio-reduction of NPs. Thus, they are involved in the bio-fabrication of metallic NPs [6]. DLS is accurate for measuring the size of nanoparticles. According to [19], the size of a particle is related to the scattering time, as small-size molecules scatter faster than larger-sized molecules. The manipulation of the size of synthesized particles can be affected by the concentration of bio-reductants, which have an important influence on the shape and size of AgNPs [25]. It was also found that the extracellular formation of AgNPs depends on the dose of silver nitrate. This is in complete agreement with our results. Moreover, it was reported that the biosynthesis of AgNPs in cyanobacteria takes place both inside the cells (with particle size <10 nm) and in solution (with particle size of 1–200 nm), leading to spherical and octahedral particles over time [26]. This is again in total agreement with our results, as the range of the nanoparticles’ size was within the one previously reported. As the volume decreased, the chance to produce freely dispersed nanoparticles became much higher. As the volume increased, more nanoparticles were produced in the external solution. The smallest the nanosilver particles, the higher their antibacterial activity. This is in accordance with previous results [20]. The preferred method to determine the size of nanoparticles is DLS. [19] compared the accuracy of the DLS method to TEM (transmission electron microscopy). They clearly showed that measurements by DLS were more accurate compared to TEM results. Dynamic Light Scattering (DLS) is based on the collision of dispersed particles with solvent molecules, leading to random movement (Brownian movement) and causing light scattering. The smaller the particles are, the faster their diffusion. UV–visible plasmon resonance showed excellent congruence within the peak range characteristic of silver nanoparticles, which indicated the purity of the three samples of silver nanoparticles; the peak heights of the samples varied, corresponding to their size difference. It is important to highlight the fact that the surface of metals is similar to plasma because of the presence of free electrons in the conduction band and positively charged nuclei [18]. Therefore, metallic nanoparticles have characteristic optical absorption spectra in the UV–visible region, as the vibration modes of electrons are limited by the size and shape of the particles [18]. Indeed, [18] were able to synthesize differently sized silver nanoparticles with the same absorption band but with varying band intensities and widths, due to the varying size of the AgNPs. This is in complete agreement with our results, which showed a similar trend. The poor detection of functional groups associated with silver nanoparticles by FTIR only indicates their absence in the cell-free filtrates. The small pore diameter of the Millipore filter used allowed the removal of any aggregates or bulky materials. Indeed, [6] reviewed reports showing that cell-free supernatants contain reducing ions/moieties that transformed silver ions into their reduced nanoform. 

With regard to the antimicrobial bioassay, the pathogenic bacterium *Streptococcus* sp., which is a Gram-positive bacterium, has a wide range of hosts. It usually exhibits a high degree of resistance against antibiotics [27,28]. It was shown [29] that there is a noticeable rise in antibiotic-resistant Streptococci strains that are capable of infecting humans and animals, causing morbidity and fatalities. This rise was associated with several mechanisms including the activity of efflux pumps [30], the modifications of the antimicrobial targets by methylation of rRNA (*erm* genes) or target mutations, and enzymatic inactivation [31]. Another mechanism of multidrug resistance could be horizontal gene transfer or chromosomal point mutations caused by the excessive use of antimicrobials. Streptococcal strains also produce biofilms which are highly resistant to antibiotics. Similar, but more contagious, is the Methicillin-resistant *Staphylococcus aureus* (MRSA), which is also a Gram-positive bacterium. Through the years, this bacterium has become multi-drug resistant through both mutations and the gain of exogenous genes that have successfully converted *Staphylococcus aureus* into Methicillin-resistant *Staphylococcus aureus*, which is resistant to all β-lactam antibiotics. This, therefore decreases the efficacy of antibiotics and increases the mortality rates following infection by this [32]. Clearly, strains that are resistant to antibiotics need alternative biocontrol strategies. In that regard, biogenic silver nanoparticles represent the most favorable biocontrol alternative due to their minimal cost, possibility of massive production, lack of toxic by-products, rapid preparation, and broad-spectrum host range. Another good reason is that their biosynthesis can be simply manipulated and customized to suit the desired application, as demonstrated here. Nonetheless and most importantly, the ability of silver nanoparticles to combat multi-drug resistant pathogens, as shown also in our study, makes them plausible candidates for future antibacterial drugs. Indeed, current antibiotics can be potentiated by the addition of silver nanoparticles to increase their antibacterial potential [14]. Silver nanoparticles have both bactericidal and bacteriostatic effects against bacteria. We showed that the smallest-sized particles were the most effective at low concentration, in accordance with previous studies [33,34]. These previous works reviewed the bactericidal effect of silver nanoparticles and summarized their mechanism of action in the following steps: (1) adhesion of silver nanoparticles on surface layers, (2) AgNPs penetration in bacterial cells and damage to intracellular structures and macromolecules (protein, lipids, and DNA), (3) induction of oxidative stress by reactive oxygen species (ROS) and free radicals, and (4) modulation of signal transduction pathways. AgNPs may modulate the human immune system, promoting bacterial inhibition [35]. Silver nanoparticles have both bacteriostatic and bactericidal effect. It was shown [36] that the bacteriostatic action is more easily observed under aerobic conditions, whereas the bactericidal action is more intense under anaerobic conditions. It was suggested that AgNPs may act by decreasing the integrity of the cell membrane. Their bactericidal effect stems from their ability to penetrate the bacterial cell and disrupt its machinery and structure. The larger the nanosilver particles, the less successful they are in penetrating bacterial cells [20]. Small-size AgNPs in the range of 10 and 15 nm show more stability, biocompatibility, and enhanced antimicrobial activity [37]. A study conducted by [38] showed that silver nanoparticles synthesized by the microwave-assisted method had a size of 55 ± 10 nm, showing that they were effective against *Escherichia coli*, the Gram-negative bacterium which is hard to combat due to its outer lipid layer. The synthesis of differently shaped nanoparticles of different sizes was also reported. It should be noted that this synthetic method yielded silver nanoparticles whose size was within a range and not fixed and uniform. The manipulation of biological systems to produce nanoparticles of a specific size is difficult. Biogenic synthesis deals with a living organism, where the biosynthesis process is dependent on many factors including pH, temperature, nature of the synthesis process, precursor concentration, and bio-reductant [6]. The success in manipulating a biological system for the synthesis of nanoparticles of a certain size opens the door for many applications that need “tailored” nanoparticles. Unfortunately, studies on customized biosynthesis are scarce, especially those on species form rather pristine habitats whose microflora is underexplored and underexploited. It must be noted that it is highly needed to study unexplored organisms with potentially exceptional biosynthetic potentials and opportunities of exploitation. Our study provides novel data on the topic and describes a simple approach to reach that target. All the different independent analyses we performed confirmed the synthesis of biogenic silver nanoparticles of three different sizes and showed that those particles had different antimicrobial activity due to their size difference. Indeed, the smallest-sized biogenic silver nanoparticles were the most effective antibacterial agents. In agreement with this, a study showed that the antibacterial action of AgNPs is higher against *S.* *aureus* when nanoparticles of small size are used [39]. The inhibitory effect of nanosilver is due to the damage it causes to several cellular components, including cell wall and plasma membrane, through the generation of reactive oxygen species. This disrupts cellular respiration and permeability [40] as reactive oxygen species (ROS), such as superoxide or hydrogen peroxide, interact with lipids, proteins, or DNA, inducing cell lysis [41]. Nanosilver particles’ adverse effects also include downregulating the enzymes responsible for the bacterial secretion system [42]. Silver nanoparticles can also inhibit proteins as well as DNA replication by accumulating at the membrane and interacting with sulfur and phosphorus needed for the synthesis of DNA [6,43]. Eventually, all those damages will result in microbial cell death. Hence, antimicrobial nanoparticles can serve as a substitute for antibiotics, at least in some cases. Indeed, Ref. [29] recommended the use of nanomedicine tools as an alternative to antibiotics to counteract multidrug-resistant pathogenic bacteria. It was also shown that current antibiotics can be potentiated with silver nanoparticles. The rationale behind this is that antibiotics and nanosilver particles use different mechanisms, and their combination would prevent the development of resistance [29]. For example, rifampicin associated with silver nanoparticles increased the antibiotic bioactivity against methicillin-resistant bacteria [44]. Ref. [45] used green biosynthesized silver nanoparticles in dental applications for better biocompatibility and antibacterial impact against *S. mutans*, responsible for caries. Green biosynthesized silver caused a reduction in lactic acid and polysaccharides in bacterial biofilms [46]. In our case, the absence of the biological cyanobacterial protein corona allowed an easy and smooth penetration of the silver nanoparticles into the vicinity of the bacterial cells. Nonetheless, there is always the possibility to further develop a commercial product that can be bionically coated to allow the commercialization and application of the product. Indeed, a study [47] used proteins (Retinin or Retinin-like proteins from insects) as a base for nanocoatings that allowed the particles to be more reactive with metallic ions. This facilitated reduction, coalescence, and nucleation of the metal nanoparticles. It was shown that these bionic nanocoatings, with metal nanoparticles, achieved higher antimicrobial activity compared to pure metallic coatings. The method provided in our research is very simple and versatile and opens the door for different ways of exploitation and manipulation.

In conclusion, antimicrobial silver nanoparticles can be produced using cyanobacteria as a green platform for their biosynthesis. Biogenic silver nanoparticles proved to be effective against multi-drug resistant pathogens. They are highly recommended to be used in the future either as a supplement to antibiotics or as an alternative treatment.

## 5. Conclusions

Nanosilver particles can be biosynthesized by cyanobacteria (green synthesis). Size manipulation is achievable by controlling the concentration of the precursor. Smallest-sized nanosilver particles are recommended as an antibacterial agent. Nanosilver particles are successful biocontrol agents against multi-drug resistant bacterial strains. Future antibacterial drugs can be based partially or entirely on nanosilver particles.

## Figures and Tables

**Table 1:** Antibacterial impact of differently sized nanosilver particles.

Strain	Inhibition Zone Diameter (mm) (Nanosilver Particles Derived from a High Precursor Concentration)	Inhibition Zone Diameter (mm) (Nanosilver Particles Derived from a Medium Precursor Concentration)	Inhibition Zone Diameter (mm) (Nanosilver Particles Derived from a Low Precursor Concentration)	Inhibition Zone Diameter (mm) of Control Disc (chloramphenicol)
*Staphylococcus aureus* (MRSA)	11 ± 2	13 ± 1	16 ± 1	28
*Streptococcus* sp.	12 ± 2 ± 2	14 ± 1	18 ± 1	25

**Table 2:** Antimicrobial Bioassay Determning MIC/MBC in μg/mL.

Bacteria	Smallest		Medium		Largest	
	MIC	MBC	MIC	MBC	MIC	MBC
*Streptococcus* sp.	1	1.5	2.5	2.5	4	4.5
(MRAS) *Staphylococcus aureus*	2	2.5	3.5	3.5	6	7

## Data Availability

All data generated or analyzed during this study are included.

## Supplementary Materials

The following are available online at https://www.mdpi.com/article/10.3390/antibiotics11081003/s1, Figure S1: FTIR from middle-sized AgNPs; Figure S2: FTIR from largest sized AgNPs
