# Supplementary material for: Multidrug-Resistant Bacterial Pathogens and Public Health: The Antimicrobial Effect of Cyanobacterial-Biosynthesized Silver Nanoparticles
Source: Antibiotics (Basel). 2022 Jul 26;11(8):1003. doi: 10.3390/antibiotics11081003 (PMC9330853; doi:10.3390/antibiotics11081003)
Supplement: Supplementary file 1 [file antibiotics-11-01003-s001.zip › antibiotics-1799790-supplementary.pdf]

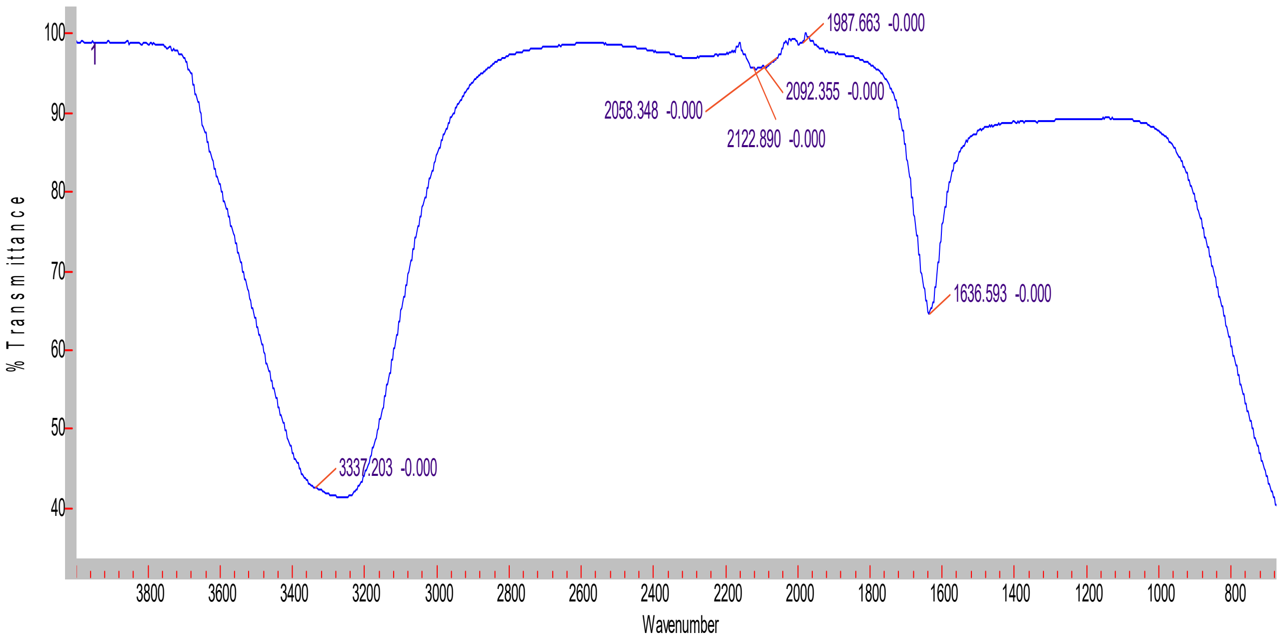

Figure S1 :FTIR from middle- sized AgNPs. X-axis denotes wavenumber, Y-axis % Transmittance

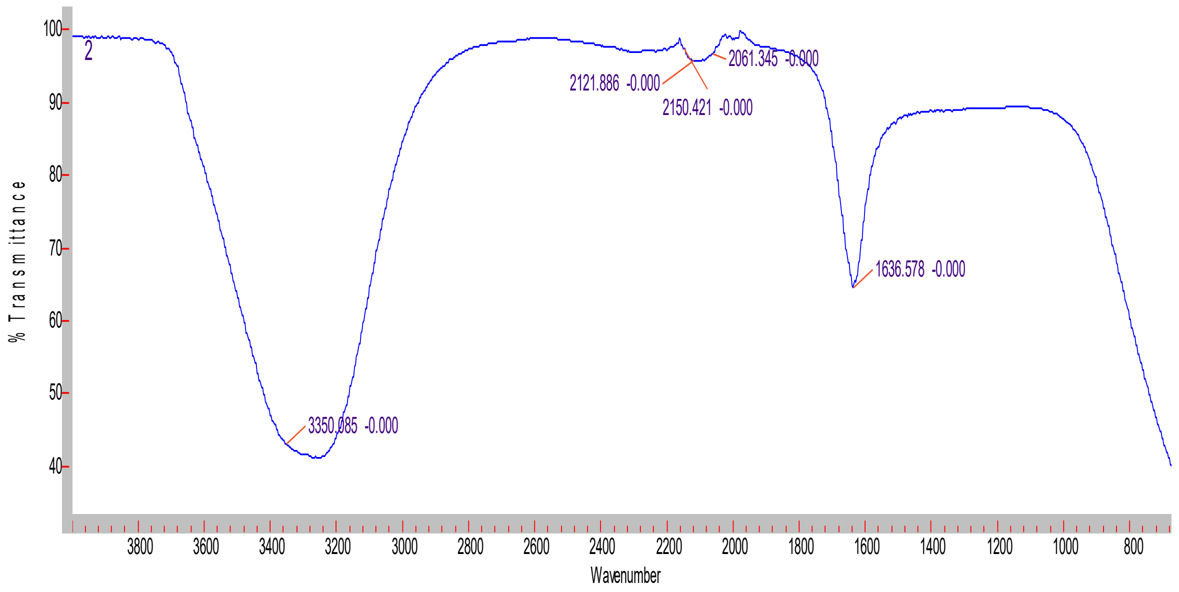

Figure S2 :FTIR from largest sized AgNPs. X-axis denotes wavenumber, Y-axis % Transmittance
